# New developments in crystallography: exploring its technology, methods and scope in the molecular biosciences

**DOI:** 10.1042/BSR20170204

**Published:** 2017-07-04

**Authors:** John R. Helliwell

**Affiliations:** School of Chemistry, University of Manchester, M13 9PL, U.K.

**Keywords:** biochemical techniques and resources, crystallography, case studies

## Abstract

Since the Protein Data Bank (PDB) was founded in 1971, there are now over 120,000 depositions, the majority of which are from X-ray crystallography and 90% of those made use of synchrotron beamlines. At the Cambridge Structure Database (CSD), founded in 1965, there are more than 800,000 ‘small molecule’ crystal structure depositions and a very large number of those are relevant in the biosciences as ligands or cofactors. The technology for crystal structure analysis is still developing rapidly both at synchrotrons and in home labs. Determination of the details of the hydrogen atoms in biological macromolecules is well served using neutrons as probe. Large multi-macromolecular complexes cause major challenges to crystallization; electrons as probes offer unique advantages here. Methods developments naturally accompany technology change, mainly incremental but some, such as the tuneability, intensity and collimation of synchrotron radiation, have effected radical changes in capability of biological crystallography. In the past few years, the X-ray laser has taken X-ray crystallography measurement times into the femtosecond range. In terms of applications many new discoveries have been made in the molecular biosciences. The scope of crystallographic techniques is indeed very wide. As examples, new insights into chemical catalysis of enzymes and relating ligand bound structures to thermodynamics have been gained but predictive power is seen as not yet achieved. Metal complexes are also an emerging theme for biomedicine applications. Our studies of coloration of live and cooked lobsters proved to be an unexpected favourite with the public and schoolchildren. More generally, public understanding of the biosciences and crystallography’s role within the field have been greatly enhanced by the United Nations International Year of Crystallography coordinated by the International Union of Crystallography. This topical review describes each of these areas along with illustrative results to document the scope of each methodology.

## Introduction

To first set a historical context, of especial significance is that the Protein Data Bank (PDB) was launched nearly five decades ago (in 1971) by the Cambridge Structure Database (CSD) and the Brookhaven National Laboratory [1] and which now has more than 120,000 depositions. The majority of these are from X-ray crystallography and 90% of these are from synchrotron radiation beamlines. The CSD itself was founded not so long before, in 1965, and now has more than 800,000 ‘small molecule’ depositions, the vast majority being derived from X-ray crystallography. A very recent, 2017, overview commentary on these two developments is described [2].

The preparation of suitable crystals is a well-known challenge for all areas of crystallography especially so for biological macromolecules. Indeed, as the synchrotron provided the intense X-ray beams to work with ever weaker scattering objects (i.e. the larger the object, the fewer the repeat units to actually scatter the X-rays) the trend was for crystallization to be more difficult. The making of macromolecular crystallization from an art into a systematic science has been a noticeable trend [3]. The high brilliance ‘third generation’ synchrotron sources greatly reduced the required crystal size into the microns range and formed an excellent synergy with the microlitre and nanolitre sized crystallization droplets trend.

Neutron beams provide special capabilities for where all other techniques fail, notably in discerning the positions of hydrogen atoms (as deuteriums, which is required to turn the negative scattering cross-section of hydrogen into the positive value of deuterium). But the magnitudes of neutron scattering cross sections are low and crystals always had to be especially big. New technology (detectors and improved placement of instruments with respect to the neutron source) and attention to the diffraction physics optimization (use of white beam ‘Laue diffraction and use of long wavelengths to enhance the scattering cross section) has led to a growth in this field in Europe, Japan and U.S.A. The complementarity of X-rays and neutrons in the biosciences has been a long sought goal for providing as complete a biological structure as possible and major strides have been made.

The selection of examples in the latter portion of this review is obviously a personal illustration given that there are over 120,000 crystal structures in the PDB to choose from and many relevant-to-biology chemical ligands held as crystal structures in the CSD. The selection I have made though follows reasonably generic research themes. These themes also illustrate the challenges in the field and how they might be surmounted with new technologies such as with synchrotron upgrades, electron microscopy developments and the relatively new ‘kid on the block’ the X-ray laser.

Firstly, in ***molecular recognition*** can we relate 3D structure to the thermodynamics of binding and does this need molecular dynamics simulations beyond the ‘static crystal structure’?
Secondly, ***chemical catalysis*** is a huge part of the biosciences notably how we understand the workings of enzymes but being able to predict catalysis is an extremely difficult, not yet solved, challenge.
Thirdly, in accurately obtaining a crystal structure identifying the chemical identity of metal ligands is often vital; anti-cancer ***metal containing drugs or medical imaging agents*** is an area of research where crystallography can help definitively to characterize and improve them.
Fourthly, the ***natural world around us*** presents challenges to our understanding at the molecular level; why lobsters are blue and why they turn red on cooking is an example of a phenomenon known to everyone. Whilst most of our scientific research work is rather abstract to the public and schoolchildren these coloration phenomena are visible to the eye and also arouses the curiosity of people!

In any area of science, including the science of crystal structures, progress depends on our skills in posing insightful questions and hypotheses as well as seeking to predict new phenomena.

## Technologies and instrumentation

### Synchrotrons

The first dedicated synchrotron X-ray source, the U.K.’s SRS at Daresbury, came on line in 1981, with the plans for its use in protein crystallography laid out in the mid to late 1970s. These ideas and plans were controversial as measured by the previous work. Where these have taken us, 40 years later, has indeed proved highly beneficial. A summary of how all this worked out, from the perspectives of some of the most senior scientists in the field from America, Asia and Europe is provided here [4].

**Figure 1:**
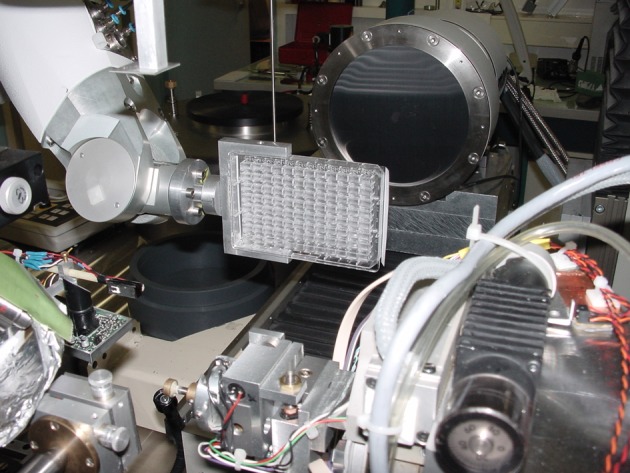
An example of synchrotron macromolecular crystallography X-ray diffraction data collection These days one does not even have to harvest one’s crystal from a crystallization tray [7]! The robotic arm is visible from left holding the crystallisation tray. Reproduced with the permission of the author, Dr Jean-Luc Ferrer and reprinted from the publication Jacquamet et al. [7] with permission from Elsevier.

Indeed, the vast majority of X-ray crystal structures in the PDB are based on synchrotron data. A major part of this was the transition to high brightness third generation SR sources. The first of these was the European Synchrotron Radiation Facility (ESRF). It was a very exciting and yet very challenging prospect in the 1980s. Would the samples, especially protein crystals, withstand the ultra-intense X-ray beams? Would the X-ray monochromators withstand the photon beam heat loads onto them? How would the detectors cope with the X-ray diffraction pattern count rates? These various challenges were addressed firstly theoretically then as pilot projects and then as routine operations. The U.S.A.’s Advanced Photon Source (APS) and Japan’s Spring 8 (Super Photon Ring 8GeV) operations followed soon after the ESRF started its operations. APS introduced top up of the electron beam, a significant development for intensity stability of the beams delivered from the X-ray optics. The work horses of the ESRF, APS and Spring 8 were (and are) X-ray undulators, which provided incredibly intense X-ray beams. Also, by varying the magnet gap, these undulators provided tunable X-ray wavelengths too. Overall massive changes in capability over the second generation SR source beamlines came about. Thus, much smaller focussed beams became possible and microcrystal samples became routinely amenable to measure X-ray diffraction data from. All the X-ray diffraction data collections we did at SRS such as optimized anomalous dispersion element identification or phasing, large unit cells and high resolution measurements were now routinely possible with much smaller samples and much shorter measuring times. Eventually, travel to the source became a chore for users and remote access ‘Fedex style’ operations commenced in 2005 at the Stanford Synchrotron Radiation Laboratory (SSRL), Stanford, U.S.A. [5]. Handling of samples using robotics has become quite a regular occurrence such as the ‘MASSIF’ (the *Massively Automated Sample Selection Integrated Facility*) at ESRF [6]. For the most fragile crystal samples, it is important to avoid extracting the crystal sample from a crystallizing tray and *in situ* X-ray diffraction of samples in the crystallization tray was born ([7], see Figure 1). Such an approach was also commercially introduced for home Lab sources [http://www.natx-ray.com/products/G-Rob_2D.html; http://www.oxford-diffraction.com/pxscanner.php]. *In situ* diffraction, as it is known, is available as well now at the Swiss Light Source [https://www.psi.ch/sls/pxiii/] and at Diamond Light Source [http://www.diamond.ac.uk/Beamlines/Mx/VMXi.html].

The detectors used for synchrotron X-ray diffraction successively changed from photographic film to TV systems, to imaging plates to CCDs and finally to pixel detectors. Datasets that were unimaginable in the home Laboratory, took ‘only’ an 8 h shift to measure at SRS and in turn were measured in minutes or seconds at ESRF. Samples which were 100 µm in size became 10 µm. The PDB depositions accelerated in number [8]. A very recent, breathtaking, development was a study which included more than 800 PDB depositions underpinning a single publication and which extended the sensitivity of the protein crystallography method to being able to see more weakly bound ligands in electron density maps [9].

A fourth generation of even lower emiitance, higher brilliance, SR source beams became possible due to a pioneering machine lattice design at MAX Lab, Lund, Sweden (http://www.vr.se/download/18.61c03dad1180e26cb8780006182/maxiv.rapport5.2006.pdf). Thus, MAX IV was conceived and entered operation recently. A major upgrade is underway at ESRF called the Extremely Brilliant Source (EBS). The streaming of sub-micron crystal samples passed these incredibly intense beams, a data collection protocol originally developed at the Linac Coherent Light Source (LCLS) at Stanford, U.S.A. (the first X-ray laser), is now harnessed at ESRF and PETRA III (Positron-Electron Tandem Ring Accelerator) [10]. The application to biological macromolecular complexes that associate (‘crystallize’) into clusters only very weakly are now coming into feasibility for X-ray diffraction. The term ‘crystal’, involving only a few unit cells’ in such a cluster, is maybe not even appropriate!

### X-ray lasers

With the incredible development of SR sources still in energetic advancement, what is the different capability possible with an X-ray laser? Firstly, let us discuss some background. The ‘Free Electron Laser’ (FEL) research in accelerator physics goes back a long way. For X-ray crystallographers great interest, pro and con, arose when it became clear that the FEL emission could extend into the X-ray range and with potentially colossal increases in peak brilliance. These gains arose, partly, from the much shorter time length of the FEL pulse. Whereas synchrotron bunches last 100 ps or so with an FEL a bunch lasts around 10 fs. But a further factor to increase the peak brilliance is the self-amplified spontaneous emission (SASE) effect [11]. The Stanford Linear Accelerator Center (SLAC) had a long LINAC, from high energy physics research days, that could be readily adopted to construct the first X-ray LCLS. LCLS has been the global focus for numerous X-ray diffraction and spectroscopy frontier research experiments these last 5 years. The last 2 years has seen the start of operation of the X-ray laser ‘SACLA’ in Japan (http://xfel.riken.jp/eng/). Of especial relevance to this topical review are e.g.:

- Radiation damage free room temperature protein crystal structures, the X-ray damage processes being slower than the initial X-ray exposure time [12].
- Femtosecond time-resolution X-ray crystallography following light flash for studies of the photosystem II ‘S states’ is an area of very active study by multiple groups worldwide and is a long standing ‘grand challenge’ [13].
- Routine use of micron to sub-micron crystals of especial relevance to those that have not crystallized readily such as membrane bound proteins in general and GPCRs (guanine-protein coupled receptors) in particular.

If there was a breakthrough moment regarding application of an X-ray laser to biological crystallography, we can define this as when the first new structural information was obtained; reference [14] used molecular replacement of an existing structure but yielded new structural information from the difference electron density map. The first fully *de novo* protein crystal structure has not yet been reported but successful feasibility studies with a range of phasing methods have been made [15].
In terms of the machine development, the European X-ray laser ‘XFEL’ based in Hamburg (http://www.xfel.eu/) is due to come online within a year or so and offers considerable increases in the rate of pulses delivered. The LCLS II upgrade is a path towards even more intense individual pulses over the LCLS I and is aiming for first light in 2019. LCLS II’s X-ray beam at the sample is believed to be sufficient for single molecules to yield measurable X-ray diffraction [16].
At the X-ray laser the ‘cost per experiment’ is considerably higher than at the synchrotron as there are fewer instruments served. This has perhaps delayed some countries developing their own X-ray lasers. An exception, besides USA and Japan, is the Swiss FEL (https://www.psi.ch/media/swissfel) which is to commence operation imminently i.e. during 2017.

An extensive summary of the field has been published recently [17]. A particular category of research benefitting from the X-ray laser are membrane proteins; membrane proteins structures solved by SFX (Serial Femtosecond Crystallography) with XFELs are described in e.g. reference [15].

### Home X-ray sources and commercial suppliers

Many protein crystals do diffract well enough for a home laboratory X-ray diffractometer. As a result research students and undergraduate final year project students have a ready access to such a local facility so as to learn how to measure good X-ray data. Progress with research projects is also made in an expeditious manner, free of a beamtime approval committee (although blocks of time are now allocated to research groups at synchrotrons to allow a more continuous user operation). The home laboratory technical capabilities have also steadily improved both with respect to X-ray source intensity at the sample and the quality of detector for better and better measurements. The commercial suppliers of home laboratory diffractometers deserve great credit for those developments.

It is also in the domain of electronic area detectors for crystallography that has seen a great deal of R&D activity by the commercial suppliers. These have evolved through several generations such as the Enraf Nonius ‘FAST’ TV detector, the MWPC (e.g. Area Detector Systems Corporation (ADSC) and Xentronics), the image plate (e.g. from Rigaku or MAR Research), CCDs (MAR Research, ADSC, Bruker, Rigaku or Oxford Diffraction) and now the pixel detectors from Dectris (see e.g. https://www.dectris.com/white-papers.html). These devices have not only been available for home labs but installed at synchrotron beamlines.

### Neutron sources and instrument developments

In pinning down details of the hydrogen atom positions notably of ionisable amino acids or bound water hydrogens, use of X-rays or nuclear magnetic resonance (NMR) may fail. For neutron macromolecular crystallography, a major breakthrough was the neutron sensitive image plate. Before this, the low fluxes of neutron sources meant that the technique was restricted only to pioneering developments. The establishment of image plate-based instruments in Tokai and in Grenoble in the mid-1980s changed radically the capabilities for user experiments [18]. Soon after the USA LANSCE facility [19] with a different approach using a spallation neutron source again expanded options. The USA Oak Ridge National Laboratory (ORNL) Macromolecular Neutron Diffractometer (MANDI) instrument and then IMAGINE [20] provided important further capacity as did the JPARC instrument [21]. Upgrades to the Grenoble instrument have followed. Monochromatic nMX instruments D19 (https://www.ill.eu/instruments-support/instruments-groups/instruments/d19/description/instrument-layout/) and BIODIFF in Munich (http://www.mlz-garching.de/biodiff) have also made strides forward with routine user access. The European Spallation Source (ESS) in Lund, Sweden has in its first tranche of instruments the nMX instrument, a highly versatile and exciting new instrument with robotics capability planned [https://europeanspallationsource.se/macromolecular-diffractometer-0]. The beamtime review committees for these ‘nMX’ instruments look for all avenues for solving a research challenge to have been tried (X-rays or NMR or electrons) before approving a project’s neutron beamtime. Bluntly put the failure of the usual probes is to be documented before use of neutrons is approved. There is now a book on the whole topic of nMX [22].

### Electrons as probe; CryoEM and electron crystallography

Experiencing a new and incredibly effective spurt of development is the use of cryo electron microscopy (CryoEM). Recent technological advances, such as the introduction of the direct electron detector, have transformed the field of cryo-EM and thereby the landscape of molecular and cellular structural biology. A simple measure of this is the rate of depositions at the Electron Microscopy Data Bank (EMDB); a recent report [23] states “*Over 1000 entries were released in 2016, representing almost a quarter of the total number of entries (4431). Structures at better than 6 Å resolution are one of the fastest-growing categories, while the share of annually released tomography-related structures is approaching 20%. The use of direct electron detectors is growing very rapidly: they were used for 70% of the structures released in 2016, in contrast to none before 2011. Microscopes from the company ‘FEI’ (https://www.fei.com/life-sciences/structural-biology/) have an overwhelming lead in terms of usage.”* A review of the potential uses, and challenges facing single-particle electron microscopy as a tool for structure-based inhibitor design is described in [24].

Electron crystallography is also developing and is utilizing molecular replacement [25]. The strong scattering interaction of electrons as probe allows use of sub-micron crystals. The potential for the use of electrons includes being sensitive to the presence of hydrogens; this example is from chemical crystallography [26].

### The effects of irradiation: a comparison of X-rays, electrons and neutrons as the probes used

A major improvement in capability for the biosciences was the widespread use of cryocrystallography using synchrotron X-rays since the sample can withstand a much larger X-ray absorbed dose at cryo temperatures [27]. This was with the disadvantage of the temperature for the measurements being far removed from the requirement for bioscience structural results to be close to physiological temperature. Apparently minor structural changes occurred between cryo versus room temperature crystal structure results. A key step in any case was to be sure, at any measurement temperature, of what the absorbed X–ray dose by the protein crystal sample actually was [28]. It was immediately clear that significant structural changes occurred with X-ray absorbed dose at room temperature [28] or cryo temperature [29,30]. It is relatively early days in the widespread application of cryo EM but investigations will need to be made of both the impact of cryo temperature and electron irradiation on structure.

How can such anxieties of irradiation effects and use of temperatures well away from physiological be avoided? Neutron crystallography is one method since neutrons are basically undamaging to the sample and measurement temperatures can be at room temperature too [18]. Nave and Hill [31] examined the possibility of reduced radiation damage for small crystals (10 µm and below in size) under conditions where the photoelectrons arising from X-ray irradiation could escape from the sample. The conclusion of this paper was that higher-energy X-ray radiation (e.g. 40 keV) could offer an advantage as the photoelectron path length was greater and less energy would be deposited in the crystal and subsequent experiments confirmed this [32]. Another elegant solution is the X-ray laser and where the promptness of the 10 fs pulses onto a micro or nano crystal yields a diffraction pattern before any X-ray radiation damage can occur and also the diffraction is measured at room temperature. As a specific example the photoreduction of the manganese ions in the manganese calcium containing oxygen evolving complex structure within photosystem II has harnessed this benefit of the X-ray laser approach [12]. The X-ray laser method of course requires many samples being used to make a dataset.

### Crystal structure determination, refinement and validation of biological macromolecule X-ray crystal structures

The phase problem in crystallography is a quite famous one in mathematical physics. In biological crystallography there are so many developments, incremental perhaps, but taken together have revolutionized the field that phasing is nowhere near the barrier that it once was. Software packages embodying these many developments are the mainstay of the life of the modern day protein crystallographer. A complete and unique recent overview of phasing in crystallography can be found in the treatise by Carmelo Giacovazzo [33].

After structure determination, which means ‘solving the crystallographic phase problem’, a structure is refined. Model refinement programs and graphics display programs offer diagnostics for validation using ‘molprobity’ routines [34] and interpreting as many of the difference Fourier map (unexplained) peaks as possible. A highly logical development initiated by Deiderichs and Karplus [35] is to process the diffraction data into the region where very few if any diffraction spots are visible. The model is refined to see if further improvements in the model accrue. This is then done in an iterative way until convergence is reached. This method avoids arbitrary cuts in the diffraction data.

The PDB has a service (https://validate-rcsb-1.wwpdb.org/) now which offers a ‘pre-validation report’ and which is of great value to authors, much like the IUCr CheckCIF utilized by small molecule (chemical) crystallographers [http://checkcif.iucr.org/]. The message from the PDB to the user states “*Welcome to the wwPDB validation system. This server performs the same validation as you would observe during the (PDB) deposition process. This service is designed to help you check your model and experimental files prior to start of deposition*.”

The macromolecular model is then deposited by the researcher at the PDB. There are regional places to interact with namely in the USA with the RCSB (Research Collaboratory for Structural Bioinformatics), in Europe with PDBe and in Asia with PDBj. Helpful utilities exist at these sites such as the validation report utility. When ready, the data files can be ‘submitted’, a formal step at which a PDB code is issued. A full validation report [36] is then provided for submission with an article to a journal.

Whilst a vital part of modern crystal structure article peer review unfortunately in many situations the PDB summary validation reports are insufficient to pinpoint the validity of an article’s claims and models based on specific electron density interpretations. The concern over the occurrence of ‘bad apples’ in structural repositories and the need for action from editors of journals has been actively made [37,38]. A very recent, extreme, case of incorrect publication of protein structures and admission into the PDB, is described here [39]. From a recent informal polling of colleagues, a wide variety of journal editors forward to manuscript authors any referee’s requests for structure factors and model coordinates to allow full validation of an article’s narrative. These requests by referees for access to the underpinning data for a submitted article are now growing in number. Unfortunately there are instances where either a journal or author has refused access to the data by the referee, but which is basically research malpractice. Clarity on data refereeing is improving and a summary policy for Springer and Nature can be found here at http://www.springernature.com/gp/group/data-policy/policy-types; ‘Type 4’ refereeing of the article’s data is defined as the most rigorous. In crystallography this level of rigour is done for chemical crystallography articles and their underlying atomic co-ordinates and processed diffraction data in Acta Cryst C. Also the CSD makes available on referee request access to deposited coordinates and structure factors underpinning a submitted article.

It may also be necessary for the referee of an article to require the reprocessing of the raw diffraction data. This opportunity has hardly ever been available to referees due to the large size of these datasets preventing their deposition. However, there is now a digital storage revolution which crystallography is beginning to take advantage of and a raw diffraction dataset is now possible to be archived, formally registered with a digital object identifier (‘doi’) and subsequently accessed for independent analysis with important benefits [40].

An independent initiative now running for many years is the PDB_REDO project [41]. This provides a re-refined i.e. new coordinates set for each and every PDB deposit. It also offers a useful server to assist the depositor, before deposit, to look at the PDB_REDO version of their current cycle of model refinement.

In another aspect, in looking at the figures in a publication, authors need to more carefully scrutinize these than perhaps they realize, in particular the display of a protein structure and its ligand binding. Of course, the immediate pre-requisite is to have confidence of the reader in the fitting of the ligand to the electron density map by the author ([42], and references therein). Assuming that is satisfactory then the depiction of non-covalent interactions needs to be to a ‘proper precision’ of any displayed distances [43]; two decimal places may well be inappropriately optimistic and sometimes even integer values only should be assumed! A website tool for providing error estimates on atomic coordinates is now available, from which such distances are calculated and specific types of examples are provided [44]. Obviously the covalent bond distances within a protein and its ligands will be ‘dictionary values’ and therefore relatively more precise and they are constrained to such values during protein model refinement.

Chemical crystallography style of validation is summarized recently here [45], and I quote “*A (crystal) structure report should therefore not only include the author’s interpretation of the experimental data but also all details of the analysis, including the primary diffraction data. All non-standard procedures, including the applied refinement constraints and restraints, should be detailed and all non-standard results reported and discussed. Only then, proper evaluation by referees, authors and users of the reported science will be meaningful and possible.”* The same good practice should apply in biological crystallography.

## Exploring examples of results

### *Molecular recognition* and relating 3D structure to the thermodynamics of ligand binding

A priority topic for molecular research is to seek to relate the 3D structure of a receptor and ligand determined from crystallography to the thermodynamics of binding via the Gibbs free energy change. A dependent question is:- does this need molecular dynamics simulations beyond the ‘static crystal structure’ to see a good correlation? There are two different aspects to this. The first is in the pharmaceutical drug discovery context where companies seek to screen their libraries of compounds against a receptor target of health interest [46,47]. This requires an estimation of binding energy and thereby allowing a ranking order of likely lead compounds. The alternative to prediction is to undertake in a systematic way as many ligand bound crystal structures as possible; thus the fragment screening approach uses this [48]. A second aspect is where there is a family of similar ligands for a given binding site; can the measured binding energy differences from microcalorimetry measurements be understood from the 3D structure? An example of this involved examination of the differences between mannoside and glucoside binding to the plant lectin concanavalin A where detailed crystallography and isothermal microcalorimetry data were available thus making an excellent model system [49]. To reach an agreement with the microcalorimetry, it was found necessary to include molecular dynamics simulations to add in the effect of the transient hydrogen bonds. Another theme for this important topic of relating structure to thermodynamics of ligand binding is in the validation, and rechecking, of ligand bound crystals structures in the PDB. Thus, Lamzin and colleagues [50] have developed and tested a methodology where ligand binding energies can be calculated and thereby seek to expose cases in the PDB where over enthusiastic interpretation of difference electron density maps might have been made by a depositor.

### *Chemical catalysis* and the workings of enzymes

Amongst the first protein crystal structures, after the globins, were of enzymes. One might imagine therefore that following the paradigm that from 3D structure we can predict function, we can expect then that the reaction rates of enzymes could be predicted. One of the pioneers of this field of enzyme crystallography was David Blow (1931–2004); he shared the Wolf Prize in Chemistry in 1987 for this research along with David Phillips (1924–1999), who determined the first enzyme crystal structure, that of hen egg white lysozyme. The Wolf Prize 1987 citation stated “*for their contributions to protein X-ray crystallography and to the elucidation of structures of enzymes and their mechanisms of action”.* Despite this obviously major accomplishment, in David Blow’s overview article in 2000 [51], he lamented that a prediction of an enzyme’s reaction rate was still not possible, in effect defining it as one of today’s continuing Grand Challenges for science [52]. This seems a harsh assessment in that qualitatively one can now see directly for example that large substrate enzymes are much slower than small substrate enzymes. There has been strong interest in developing X-ray crystallography to achieve a mechanistic view including enzymes and is reviewed here [53]. One approach is that an enzyme’s reaction rate is deliberately slowed down, even stopped, by working with a designed mutant of the enzyme, again illustrating that, if not exactly a prediction of a specific reaction rate, this is a deliberate and successful alteration of enzyme reaction rate. Such an example involved use of a flow cell to diffuse substrate directly into an enzyme crystal for the time-resolved Laue crystal structure analyses of the enzyme hyroxymethylbilane synthase [54]. In that experiment the enzyme’s reaction rate was deliberately slowed down by working with a designed mutant of the enzyme, again illustrating that, if not exactly a prediction of a specific reaction rate, this was a deliberate and successful alteration of that. A very interesting methodological development is the introduction from flash photolysis with lasers into the X-ray crystallography domain of the Hademard measuring sequence [55]. This deliberately sets up a time sequence of measurements, shorter than a measuring period and thereby allows extraction of finer time resolution details. The wide applicability of this to both weaker X-ray sources and to neutron sources would widen the scope for such studies. There are many closely related developments in time-resolved diffraction. Applying cryo quench techniques the freezing time is minimized with single molecular complexes, and which are studied by cryoEM [56]. A major advance in biological chemistry has been the application of synchrotron radiation to determine biological crystal structures at atomic resolution to give as clear details as possible of reactants and products [57]. The application of neutrons in biological chemistry to determine any missing protonation and bound water details is under active development and wide application [58].

Where does the future lie? The quest for X-ray laser diffraction of single molecules remains an objective and that, like cryo EM, gets away from the need for a crystal and its packing restrictions. As with the previous section molecular dynamics calculations are increasing their scope not least reaching a total time length of simulation up to the microsecond level for a protein; these simulations can be linked to the whole diffraction pattern analysis for a protein crystal X-ray diffraction’s Bragg reflections and the diffuse scattering [59].

#### Metal containing drugs and medical imaging agents

##### Harnessing two X-ray wavelengths

**Figure 2:**
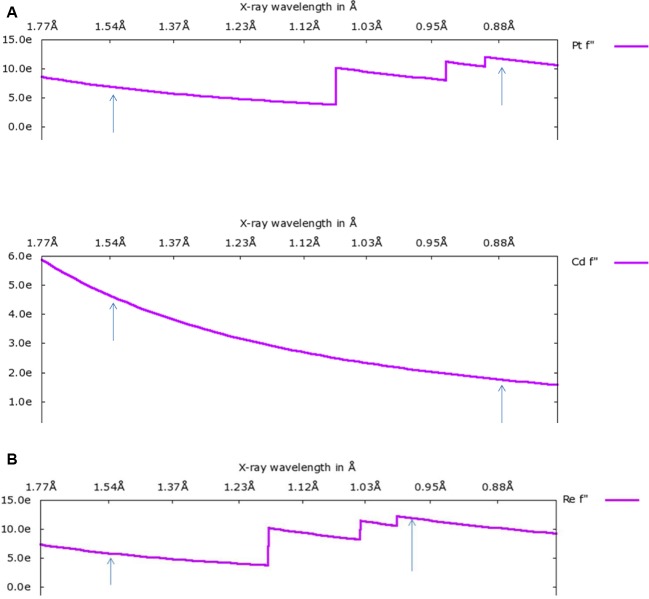
Analysing metal atom binding to biological macromolecules There is a good utility of using two synchrotron X-ray wavelengths for element discrimination or enhanced sensitivity to lower metal atom occupancy; the variation in f “ is shown for simplicity. The arrows indicate the X-ray wavelengths used in measuring the diffraction datasets in the studies [60,61]. There is a variation also in f ‘ which must be allowed for in refining a metal atom occupancy. Discriminating between two elements in the cases shown (**A**) Pt versus Cd harnessed in the crystal structure analysis of encapsulating the anti-cancer drug carboplatin in a (ferritin) protein shell nanoparticle; ferritin crystals require cadmium ions for their crystallization but the range of metal occupancies leads to either Pt or Cd being plausible interpretations at binding sites unless two X-ray wavelengths are used [60]. (**B**) Enhancing the sensitivity of crystal structure analysis to weak metal binders two X-ray wavelengths give a comparison of peak height changes between the two in the case shown for rhenium, see also Table 1. Rhenium is used as a chemical model for technetium with a view to designing improved breast cancer imaging agents [61]. These figures are prepared using http://skuld.bmsc.washington.edu/scatter/AS_form.html. Copyright Ethan A Merritt ^©^1996–2011 to whom I am very grateful.

This field is being increasingly investigated both for where the metallated agent binds and how their delivery might be optimized using bionanoparticles. We illustrate this theme with firstly the platins and secondly the technetium imaging agents. Technetium is of course an unstable element, but is possible to investigate its chemistry via its close chemical relative, rhenium. In terms of technique, it has proved fruitful to harness two X-ray wavelengths, in different ways, as we describe below for both themes. The utility of using two synchrotron X-ray wavelengths is for element discrimination (Figure 2A) or enhanced sensitivity to lower metal atom occupancy (Figure 2B and Table 1).

**Table 1:** The increase in anomalous difference electron density map peak heights in the Cu Kα and Diamond Light Source diffraction data due to the optimized diffraction wavelength; three rhenium binding site examples are shown so as to illustrate high, medium and lower occupancy rhenium atoms (Brink and Helliwell [61]).

Residue	Cu Kα (λ = 1.5418 Å)	DLS (λ = 0.9763 Å)
	Re *f* “ = 5.9	Re *f* “ = 12.1
His 15	19.3	43.1
Asp 52	6.9	12.9
Glu 35	4.8	12.2

**Figure 3:**
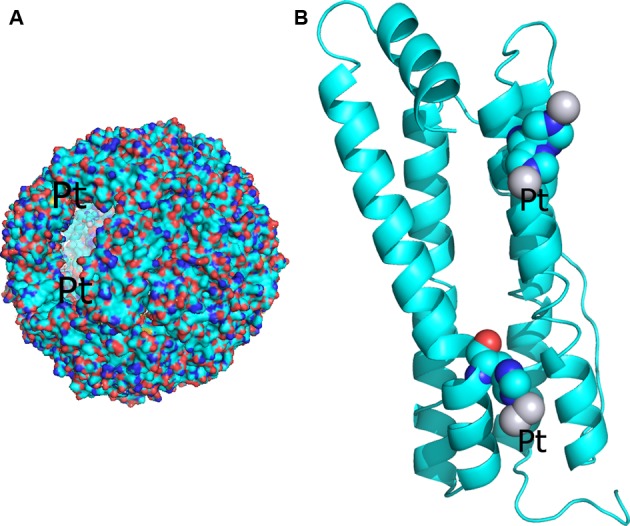
Harnessing bionanotechnology for delivering metallated anti-cancer agents Synchrotron radiation characterization of a protein (ferritin) nanoparticle encapsulating carboplatin [60]. (**A**) The whole nanoparticle and (**B**) the protein subunit with two bound carboplatin molecules; twenty four of these make up the highly symmetric nanoparticle. Reproduced with the permission of the corresponding author, Professor Antonello Merlino and reprinted (adapted) with permission from Pontillo et al. [60] Copyright (2017) American Chemical Society.

The platins is the more advanced topic area. The coordination chemistry of the platins has been extensively described in the review by Messori and Merlino [62]. The characterization of an encapsulated carboplatin in a protein nanocage (ferritin), and thereby optimizing the delivery of the drug to a target, has recently been described and used two X-ray wavelengths (Figure 2A) to distinguish between the platinum atoms and the cadmium atoms, the latter needed to crystallize the ferritin nanocage ([60], see Figure 3). A similar procedure has been also used to verify the encapsulation of cisplatin within the ferritin nanocage [63]. Within this overall theme, in another study, an iodoplatin was found [64] and this opens up a two wavelength radiation therapy possibility by harnessing the platinum K edge at 78 keV and the iodine K edge at 33 keV, each with different penetrations of course into a tumour. The study of medical radiation therapy in this way is at the research stage only at present at the ESRF medical beamline (http://www.esrf.eu/UsersAndScience/Experiments/CBS/ID17).

The rhenium binding to a variety of amino acids, besides histidine, has been discovered [61]. The use of two X-ray wavelengths in that study, including one at the rhenium L I absorption edge to optimized the rhenium signal, gave the expected increased sensitivity for the weaker occupancy rhenium binding sites (see Figure 2B and Table 1). Thus, chemical optimization to these amino acids became possible and thereby a reduced amount of rhenium for use as a medical imaging agent (as technetium) is possible.

#### Natural world around us

##### The study of marine coloration

So much of what we do with our crystal structure analyses is abstract as far as the Public and schoolchildren are concerned. One research theme obviously derived from the ***natural world around us*** is in the field of marine coloration. Indeed, why lobsters are blue and why they turn red on cooking is an example of a phenomenon known to everyone. Whilst most of our scientific research work is rather abstract to the Public and schoolchildren these coloration phenomena are visible to the eye and arouses the curiosity of people!

The key is a chemical called astaxanthin, which has the orange-red colour of a cooked lobster and how it interacts with a complex of proteins called crustacyanin which lobsters produce [65]. The reaction of astaxanthin with the protein complex gives the creature its dark blue colour. The X-ray crystal structure of a portion of the crustacyanin full ‘alpha’ complex, the beta-crustacyanin, revealed the molecular layout of the two astaxanthins bound to the protein dimer. From this it was straightforward to infer that when the lobster is cooked the protein is denatured and the astaxanthin is released from the beta-crustacyanin and reverts to its orange-red state. There was remaining however the scientific issue of what was the mechanism underlying the colour shift of free to bound astaxanthin? This is a complicated question! In a recent collaboration with physical organic chemists it was found that astaxanthin can behave as an acid [65].

When explaining these findings to the Public and schoolchildren they are keen to ask why, alive and living in the sea, lobsters are naturally a dark-blue/black colour? The best explanation, emphasizing that one doesn’t really know, is that natural selection led to this as it makes them harder to spot for predators. The main predator, besides man, is the octopus! This keen interest from the Public and schoolchildren includes the observation that, in the era of climate change, it is important to think about the delicate nature of life and the sustainability of life on the planet.

### Crystallography and sustainability

In 2014 the United Nations declared the International Year of Crystallography, organized by UNESCO with the International Union of Crystallography (IUCr). A wide ranging suite of activities took place and are described here https://www.iycr2014.org/. The biosciences were a firm part of this. The field of crystallography is contributing to each of the UN’s Millennium Development Goals (MDGs) (http://www.un.org/millenniumgoals/) and are something that every scientific field should support and indeed measure itself against [Table 2].

**Table 2:** The UN’s world’s sustainable development goals, integrated into the eight MDGs established in 2000 following the Millennium Summit of the United Nations. These were adopted by the 189 United Nations member states at the time and more than 20 international organizations.

1. To eradicate extreme poverty and hunger
2. To achieve universal primary education
3. To promote gender equality and empower women
4. To reduce child mortality
5. To improve maternal health
6. To combat HIV/AIDS, malaria and other diseases
7. To ensure environmental sustainability
8. To develop a global partnership for development

Crystallography is most obviously assisting with the UN’s Goal 6. From the earliest years of synchrotron radiation in protein crystallography structure-based drug design was considered highly relevant [66]. This has been continued very actively by SR facilities as an industrial liaison service provider on a contract by contract basis [for current examples see http://www.diamond.ac.uk/industry/, http://www.esrf.eu/Industry, https://www.albasynchrotron.es/en/industry/services, http://www.lightsource.ca/pharmaceuticals]. In addition, pharmaceutical companies have come together in two distinct ways. Firstly, they cooperated together, and in partnership with Hauptman-Woodward Medical Research Institute, established the Industrial Macromolecular Crystallographic Association (IMCA) which includes its own dedicated Collaborative Access Team beamlines at the U.S.A.’s Advanced Photon Source (APS) (https://www.imca.aps.anl.gov/) for ligand discovery towards new drugs. In another example of Pharma collaboration, the Structural Genomics Consortium (SGC) is a not-for-profit organization formed in 2004 to determine the 3D structures of proteins of medical relevance and place them in the PDB without restriction on use. The SGC operates out of the Universities of Oxford and Toronto. Over the past 5 years, the SGC has accounted for ∼25% of the global output of novel human protein structures each year and ∼40% of the annual global output of structures of proteins from human parasites. The SGC target proteins have relevance to human health and disease, such as diabetes, cancer and infectious diseases such as malaria. The SGC is an ‘open data’ partnership. All research results are published with no restriction [67]. There are also long traditions in the biological crystallography field of the studies of viruses on the one hand [68,69] and tropical diseases on the other [70]. A highly notable project is the structural genomics study of the MTb genome and its protein crystal structures (http://webtb.org/).

Re the UN’s Goal 3 crystallography has a good track record since its earliest period in the late 1910s and 1920s when William Henry Bragg had a substantial fraction of his research students being female. In crystallography, gender equality at the ‘breaking the glass ceiling level’ has occurred whereby we have seen three female IUCr Presidents namely Kathleen Lonsdale, Dorothy Hodgkin and Sine Larsen (periods of office respectively being 1966, 1981–1984 and 2008–2011). There are also female crystallographers up to Nobel Prize winner level with Dorothy Hodgkin (Nobel Prize in Chemistry 1964) and Ada Yonath (Nobel Prize in Chemistry, shared, 2009).

## Summary of trends and conclusions

Crystallography for the biosciences continues to develop vigorously alongside major accomplishments. The working of single crystal structure determination increasingly alongside cryoelectron microscopy is a major trend of researchers in structural biology. NMR and crystallography are also becoming more integrated; for example the International Union of Crystallography established a new Commission on NMR Crystallography and Related Methods in 2014 (https://www.iucr.org/iucr/commissions/nmr-crystallography). The technology of X-ray sources is advancing strongly entering a fourth generation of extremely brilliant synchrotron sources and a second generation of X-ray lasers; sample sizes reaching the single molecule size are an X-ray laser facility objective. The increasing realization that neutrons as a different probe capability to complement X-rays and electrons is growing in the community. The combination of structure determining techniques, illustrated in the crustacyanin example above of crystallography, SAXS, EM and modelling, is more and more widespread.
